# Influence of the characteristics of Japanese patients on the long-term outcomes after aortic valve replacement: results of a microsimulation

**DOI:** 10.1007/s11748-014-0499-2

**Published:** 2014-11-22

**Authors:** Tomonobu Abe, Hideki Oshima, Yuji Narita, Yoshimori Araki, Masato Mutsuga, Kazuro Fujimoto, Yoshiyuki Tokuda, Sachie Terazawa, Kei Yagami, Akihiko Usui

**Affiliations:** Department of Cardiac Surgery, Nagoya University Graduate School of Medicine, 65 Tsurumaicho, Showa-ku, Nagoya, 466-8550 Japan

**Keywords:** Aortic valve, Heart valve prosthesis, Computer simulation, Decision support techniques

## Abstract

**Objectives:**

The aim of this study was to explore the influence of the characteristics of Japanese patients on the long-term outcomes after aortic valve replacement with either mechanical or biological prostheses by means of a microsimulation.

**Methods:**

A microsimulation model was used to simulate the lives of patients living in Japan and in the United States after aortic valve replacement. The background mortality data taken from the 21st complete life table of Japan and the bleeding hazard determined from a meta-analysis of long-term results in Japanese institutions were used to simulate Japanese patients. The life expectancy, event-free life expectancy, and lifetime risk of structural valve degeneration were calculated. A sensitivity analysis for various bleeding hazards was performed.

**Results:**

Regarding the event-free life expectancy, the age crossover points between the two valve types were 64–65 and 57–58 years for Japanese and American patients, respectively. Regarding the life expectancy, the age crossover points were 88–89 and 64–65 years, respectively, for Japanese and American patients. The lifetime risk of structural valve degeneration was higher in Japanese patients than in American patients. The sensitivity analysis showed that the age crossover points were sensitive to the hazard of bleeding complications.

**Conclusions:**

The long-term clinical outcomes after aortic valve replacement were simulated with a microsimulation model. The results indicated that the age crossover points in the advantages and disadvantages between mechanical valves and bioprostheses may be higher in Japanese patients than in American subjects.

## Background

The decision regarding whether to choose a mechanical or biological prosthesis to replace diseased aortic valves is a complex problem. It generally can be considered as the tradeoff between the risk of reoperation for a bioprosthesis due to structural valve degeneration (SVD) and the risk of bleeding complications associated with long-term anticoagulation for mechanical prostheses [1]. We usually think that biological valves should be more advantageous for elderly patients, because the chance of SVD would be lower for two reasons; the shorter life expectancy and the slower degeneration of the bioprostheses.

The Japanese Circulation Society Guidelines for Surgical and Interventional Treatment of Valvular Heart Disease recommend the use of a bioprosthesis for patients older than 65 years old for the aortic position [2]. However, most of the references were from North America and Europe. Some researchers have been expressing concerns that it may be wrong to use the same recommendation for Japanese patients, because the average life expectancy is longer in Japan compared to the United States and most other developed countries [3, 4].

We also found in our previous study that the incidence of bleeding complications associated with mechanical valves was considerably lower in Japan than in other countries by performing a comprehensive systematic review [5]. Since the higher rate of bleeding complications is the most important drawback of mechanical valves, this may also influence the balance of advantages and disadvantages in the choice of prosthesis.

The aim of this study was to explore the influence of these variables on the long-term results of patients who undergo aortic valve replacement by either mechanical or biological prostheses by performing a microsimulation.

A microsimulation model is a kind of Monte Carlo simulation which simulates a representative population at the individual patient level [6]. This can offer a complementary tool to a standard analysis [7].

## Methods

**Fig. 1:**
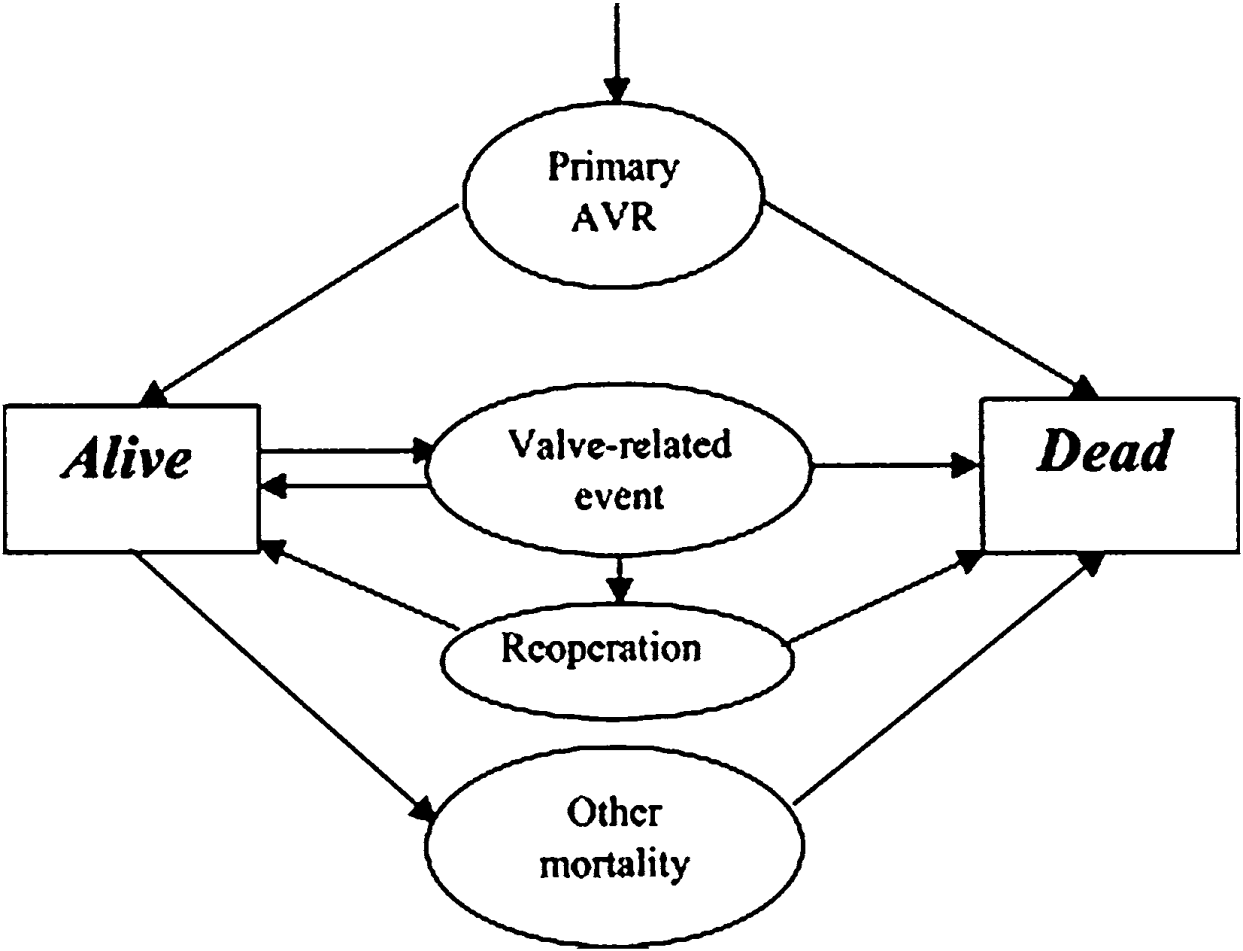
Schematic representation of the microsimulation model showing different health states of a patient after aortic valve replacement (reproduced with permission of the copyright owner. Takkenberg et al. [18]). *AVR* aortic valve replacement

A microsimulation model was used to simulate the lives of patients living in Japan and in the United States after AVR. We used a previously described microsimulation model by Rotterdam group [8]. This microsimulation model was developed to simulate the life of an age-, sex- and nationality-specific patient after aortic valve replacement either by a mechanical prosthesis or a bioprosthesis, taking into account all morbidity and mortality events and sequences of events. The details of the model have been described elsewhere [9, 10]. The basic structure of the model is shown in Fig. 1.

In brief, after aortic valve replacement, a patient either dies as a result of the procedure or survives. If the patient survives, then they remain at risk of developing valve-related events for the rest of their life. The hazard estimates for all valve-related events in the original model have been explained in detail elsewhere [8, 9, 11]. Patients may or may not survive the valve-related events with or without undergoing reoperations. The age-dependent mortality rate of each valve-related event has also been modeled. Eventually, all patients will either die due to valve-related causes or to other causes. The simulation is repeated for a large number of random patients (10,000 patients for each country, age and gender in this study) until death, so that a virtual population of country-, age- and gender-specific patients is created. From this population, average estimates of the outcome can be calculated, for example, the event-free life expectancy (EFLE), total life expectancy (LE) and lifetime event risk.

The EFLE, LE and lifetime risk of SVD for patients in Japan and the United States for various ages were calculated in this study. In this study, we changed several variables from the original model to simulate contemporary Japanese and American patients, as described below.

### Hazards used for the US population

For residents in the United States, we calculated the background mortality, which is the mortality experienced by the normal population, using American life tables derived from the Vital Statistics of the United States in 2009 reported by the Centers for Disease Control and Prevention to update the data [12]. The statistics from 1992 were used in the original model [8].

In the original model, the bleeding hazard increased with age, and was given by a Gompertz function, where lambda was −8.71. Based on this assumption, the bleeding hazard in the original model was 0.23 % per patient-year in patients aged 35 years, 1.58 % per patient-year in patients aged 60 years and 4.9 % per patient-year in patients aged 75 years [8]. We used these numbers to simulate patients in the United States.

### Hazards used for the Japanese population

For the Japanese population, we changed two probability distributions from the original model. These were the background mortality and bleeding hazard. In the present analysis we used the background mortality for Japanese patients from the 21st life table made by the Ministry of Health, Labor and Welfare of Japan based on the census conducted in 2011 [13].

We obtained the bleeding hazard of mechanical valves for Japanese patients based on our previous meta-analysis. In this meta-analysis, we included studies that reported the long-term results of valve replacement with bileaflet mechanical valves reported from institutions located in Japan between 1988 and 2010 [5]. In that study, we obtained a bleeding rate of 0.41 %/patient/year at a mean age of the mid 50s in Japanese patients who had undergone aortic valve replacement.

For the bleeding hazard in Japanese patients, we used the same Gompertz function as was used for the US population, but at a lower bleeding rate, and we substituted −9.40 for lambda. The bleeding hazard was 0.54 % per patient-year in patients aged 55 years, 0.79 % per patient-year in patients aged 60 years, 2.5 % per patient-year in patients aged 75 years and 3.6 % in patients aged 80 years based on this assumption. These numbers are compatible with the results of another meta-analysis of the results of mechanical valves in the elderly population [14].

### Sensitivity analysis for various bleeding hazards

We explored three level of hazard for bleeding events with mechanical valves as a sensitivity analysis. These were using lambda values of −9.40, −8.99 and −8.71 in the Gompertz function. The values of −9.40 and −8.71 have been already explained above. The value of −8.99 was assigned as the number in the middle of these two numbers. We performed this analysis because it is theoretically possible that the bleeding hazard derived from our meta-analysis might be somewhat lower than the true hazard because of the nature of the retrospective studies that had been included in the meta-analysis.

## Results

### Event-free life expectancy (EFLE) and total life expectancy (LE)

**Fig. 2:**
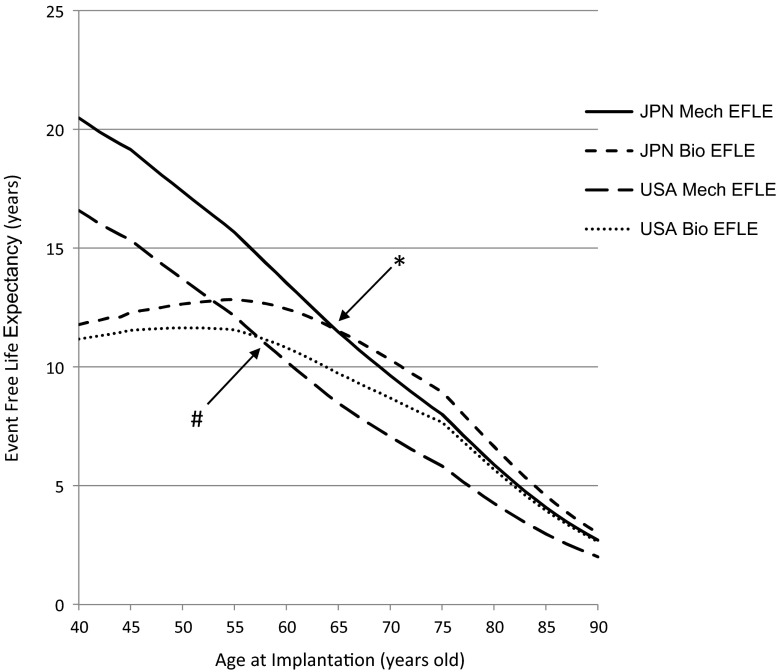
The results of a comparison of the average event-free life expectancy (years) in females after aortic valve replacement with mechanical valves and bioprostheses in Japanese and American patients. *Asterisk* and *hash symbol* indicate the age crossover points between mechanical valves and bioprostheses in Japanese and American females, respectively. *JPN* Japanese, *EFLE* event-free life expectancy, *USA* United States of America

**Fig. 3:**
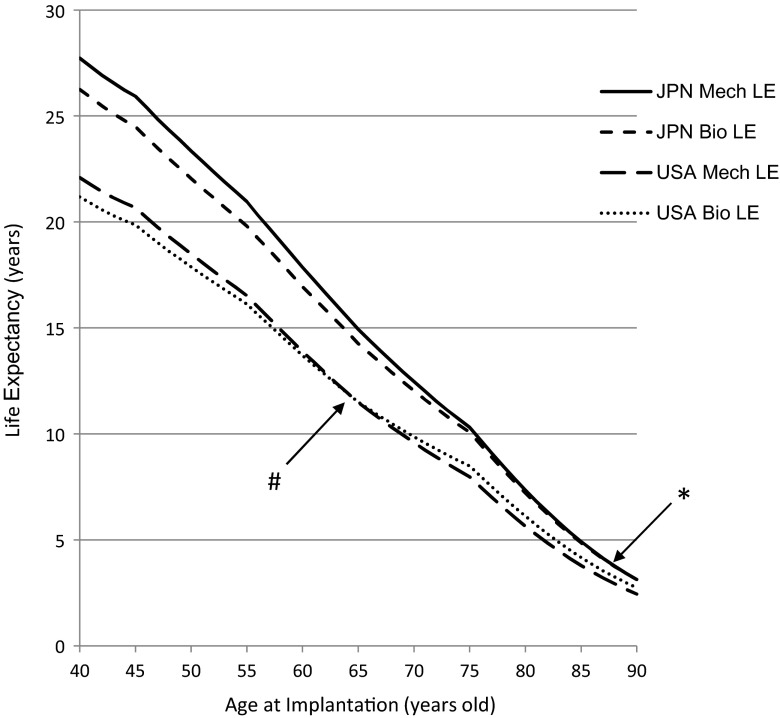
The results of a comparison of the average life expectancy (years) in females after aortic valve replacement with mechanical valves and bioprostheses in Japanese and American patients. *Asterisk* and *hash symbol* indicate the age crossover points between mechanical valves and bioprostheses in Japanese and American females, respectively. *JPN* Japanese, *LE* life expectancy, *USA* United States of America

The microsimulation model calculates the EFLE and LE following aortic valve replacement with mechanical valves and bioprostheses for patients of either sex and of different ages. The EFLE in Japanese and US females is shown in Fig. 2, and the LE values for these populations are shown in Fig. 3.

Regarding the EFLE, for a 70-year-old Japanese female, for example, the average EFLE was 9.64 and 10.30 years, respectively, after implantation with a mechanical valve and a bioprosthesis, whereas these were 7.06 and 8.69 years for an American female (Fig. 3).

The age crossover points between the two valve types were 65 and 58 years, respectively, for Japanese and American females (Fig. 2). Similarly, the age crossover points were 65 and 58 years, respectively, for Japanese and American males (figure not shown).

Regarding the LE, for a 70-year-old Japanese female as an example, the average LEs were 12.47 and 12.04 years, respectively, after implantation with a mechanical valve and a bioprosthesis, whereas the average LEs were 9.59 and 9.87 years, respectively, for an American female (Fig. 2). The age crossover points between the two valve types were 89 and 65 years, respectively, for Japanese and American females (Fig. 2). Similarly, the age crossover points were 88 and 64 years, respectively, for Japanese and American males (figure not shown).

### Structural valve degeneration (SVD)

**Fig. 4:**
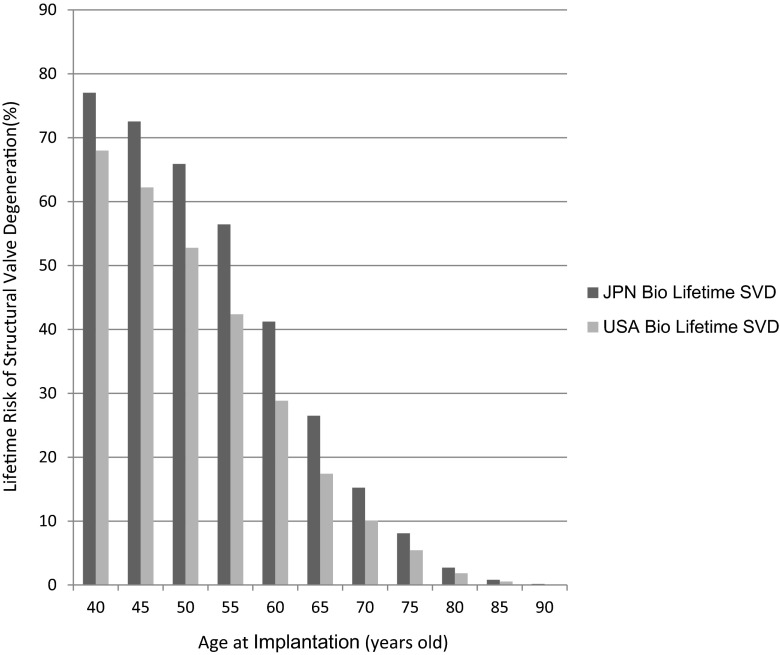
The lifetime risk of structural valve degeneration in Japanese and American females. *JPN* Japanese, *SVD* structural valve degeneration, *USA* United States of America

The lifetime risks of structural valve degeneration are depicted in Fig. 4. As shown in the figure, the lifetime risk of SVD following the implantation of a bioprosthesis decreases with an increasing age at implantation, and is about 17.42 % for a 65-year-old American female patient, and is 26.49 % for the same age Japanese female patient (Fig. 4). The lifetime risk of SVD is higher in Japanese than in Americans for all ages of implantation.

### Sensitivity analysis

**Fig. 5:**
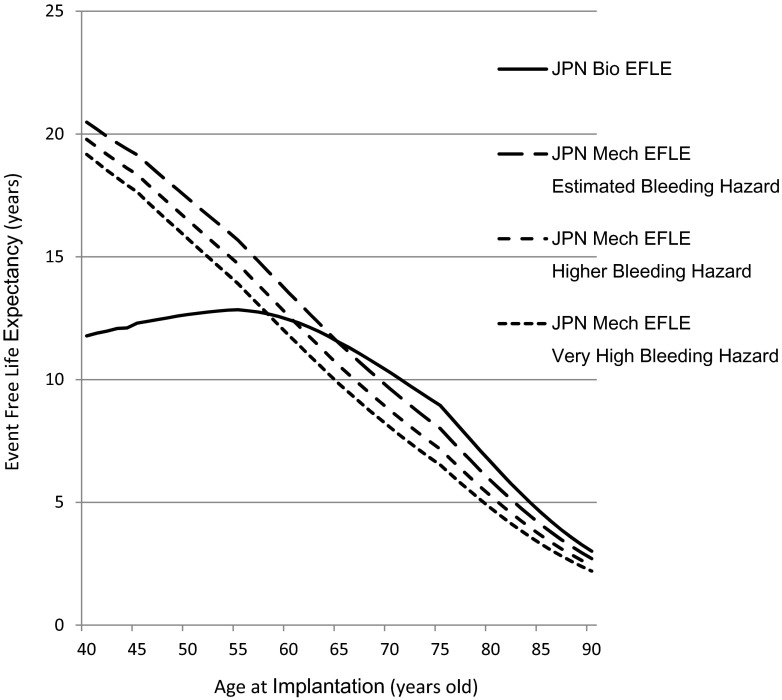
The results of a sensitivity analysis of the average event-free life expectancy for bioprostheses and mechanical valves for three different bleeding hazards associated with mechanical valves in Japanese females. Estimated bleeding hazard; lambda = −9.40, Higher bleeding hazard: lambda = −8.99, Very high bleeding hazard; lambda = −8.71. See the text for an explanation of how we chose these numbers. *JPN* Japanese, *Bio* bioprosthesis, *Mech* mechanical valves, *EFLE* event-free life expectancy

The results of the sensitivity analysis for the three bleeding hazards on the EFLE in Japanese females are shown in Fig. 5. As shown in the figure, the age crossover point between the two valves is sensitive to the bleeding hazard. The age crossover points were 65 years, as described above, for a lambda of −9.40, 61 years for a lambda of −8.99, and 58 years for lambda of −8.71 (Fig. 5).

## Discussion

The aim of this study was to explore the effects of the characteristics of Japanese patients on the clinical outcomes after aortic valve replacement with the aid of a microsimulation.

A microsimulation is a useful tool, especially when evidence is scarce and the decision to be made is complex [7]. This is indeed the case for the prosthetic valve choice in Japanese patients. There is little hard evidence supporting the prosthetic valve choice, and such data are even scarcer for the Japan population. Only three randomized controlled trials have been published worldwide, and none of these was done in Japan [15]. The prosthetic valve choice is a complex problem, because there are a variety of clinically important outcomes that can arise due to prosthesis-related complications, and the hazard changes over time after implantation. The whole remaining lifetime after surgery in each patient has to be brought into perspective.

In this study, the microsimulation model predicted the age crossover point between the valve types in Japan to be 64–65 years with regard to the EFLE and 88–89 years for the LE. These age crossover points were significantly older compared to the results of the simulations calculated for patients in the United States. The age crossover points were 58–57 years for the EFLE and 65–66 years for the LE for the patients in the United States. These results suggest that the appropriate age to recommend implantation of a bioprosthetic valve may be older in Japan compared to the United States.

As far as the EFLE is considered, the age crossover point was 65–66 years old. This is the age that the current Japanese guidelines recommend, and the recommendation is compatible with our results. However, the age crossover points were much higher when the LE was considered. Several researchers have expressed their opinion that the age recommendation for the use of a bioprosthesis should be raised from 65 years for Japanese patients based on their follow-up data [4]. Our results can be interpreted to support their opinion, because most medical professionals, as well as patients, will consider survival to be the most important clinical outcome, and more important than freedom from any valve-related events. We believe that it is advisable for clinicians to be aware that there is possibility that the choice of a bioprosthesis in relatively young patients can be harmful in terms of the life expectancy, and our results should therefore be carefully examined based on data obtained in the real world.

In this study, we explored the effect of two different probabilities. One was the background mortality in Japanese populations. Japan has been known to have long life expectancies, even among developed countries [3]. The other is the low incidence of bleeding complications, which we found in a previous systematic review [5].

Regarding the background mortality, there is little doubt about the accuracy of numbers, because these data were extracted from reliable official government sources from Japan and the United States. However, the difference in this study may be a little exaggerated as an example of the differences among Japan, North America and Europe. First, the United States has a relatively short life expectancy among developed countries, being ranked 31st in the world [3]. Canada, Australia and most western European countries have longer life expectancies. Second, it is well known that the life expectancy significantly varies among ethnic and socio-economic groups in the United States [12]. Many renowned American medical centers publishing important papers may be treating patients who belong to groups having longer life expectancies than the general population. Thus, it may be inaccurate to assume that papers from the United States are the results of patients who have the background mortality of the general population.

We found an incidence of 0.41 %/patient/year of bleeding complications in our previous meta-analysis [5] and used the lower bleeding hazard for Japanese population in this study. The lower incidence of bleeding complications may be considered contradictory to the increasing anecdotal evidence of a higher incidence of intracranial bleeding in East Asians compared to non-East Asians [16, 17]. However, we think that our results are compatible with those data suggesting Asian’s are prone to bleeding. In most of the paper included in our meta-analysis, the patients were treated at a significantly lower international normalized ratio (INR) range compared to the current recommendations [5]. We believe that this explains why patients bleed less in our meta-analysis compared to the international multicenter randomized controlled trials which mandated the same target INR range in Coumadin control among all participant countries. We also think our data are accurate, because the rate of thromboembolism, which was 1.24 %/patient/year, was the same as the results from Western countries [5]. We do not see any reason to assume that only bleeding complications were inaccurate, while the thromboembolism rates were correct.

Transcatheter aortic valve implantation has been introduced in clinical practice. This undoubtedly is a significantly different way of treating aortic valve disease compared to surgical aortic valve replacement, and may have a significant impact on the decision-making with regard to the choice of prosthetic valve. Although we were aware of this, we did not take this treatment modality into account in the current study. We believe this procedure is still immature, and it is not yet possible to estimate the accurate hazard of prosthesis-related complications and the procedure-related mortality and morbidity for a wide range of patient ages.

This study is associated with several other potential limitations that we would like to clearly state. First, we used the data from Japan for two variables. The other variables were taken from the original model, and are from a meta-analysis of papers written in English, which likely means that most of the data were from North America and Europe. It is natural to think that variables other than the two selected variables may also be somewhat different from the numbers in contemporary Japanese practice, although we did not see large differences in the incidence of other valve-related complications in our meta-analysis. We are planning to make our own original model and to update all of the variables based on a large dataset from Japanese subjects. In the meantime, the results of this study should be informative as they provide the results of a sensitivity analysis of an established model to better understand the relationships between the characteristics of Japanese patients and the balance of advantages and disadvantages for the different valve types.

Second, although this model took into account all of the valve-related complications and their sequences, a microsimulation still is a simplified model of the real world, and has its own methodological limitations, as described elsewhere [7].

## Conclusions

The long-term clinical outcomes of Japanese subjects implanted with mechanical valves and bioprostheses were simulated using an established microsimulation model with variables changed to reflect the current status in Japan. The results showed that the age crossover points of the LE and EFLE were higher than those calculated for the US general population.
